# Supplementary material for: Antioxidant Activities of Dihydromyricetin Derivatives with Different Acyl Donor Chain Lengths Synthetized by Lipozyme TL IM
Source: Foods. 2023 May 14;12(10):1986. doi: 10.3390/foods12101986 (PMC10217546; doi:10.3390/foods12101986)
Supplement: Supplementary file 1 [file foods-12-01986-s001.zip › foods-2383420-supplementary.pdf]

Supplementary material

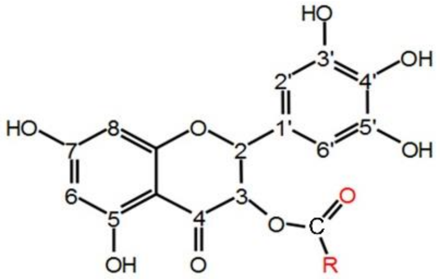

R	Compound
CH_3	3-*O*-acetyl-DHM (C2-DHM)
$(CH_2)_2CH_3$	3-*O*-butyryl-DHM (C4-DHM)
$(CH_2)_4CH_3$	3-*O*-hexanoyl-DHM (C6-DHM)
$(CH_2)_6CH_3$	3-*O*-octanoyl-DHM (C8-DHM)
$(CH_2)_{10}CH_3$	3-*O*-lauroyl-DHM (C12-DHM)

Figure S1. Structures of the acylated DHM derivatives.
